# Effects of rhBMP-2 with various carriers on maxillofacial bone regeneration through computed tomography evaluation

**DOI:** 10.1186/s40902-023-00405-6

**Published:** 2023-10-27

**Authors:** Ja In Seo, Ji Hye Lim, Woo Min Jo, Jeong Keun Lee, Seung Il Song

**Affiliations:** Department of Oral and Maxillofacial Surgery, Institute of Oral Health Science, Ajou University School of Medicine, 164 World cup-Ro, Yeongtong-Gu, Suwon-Si, Gyeonggi-Do 16499 Republic of Korea

**Keywords:** Recombinant human bone morphogenetic protein 2, Bone regeneration, Cone beam computed tomography

## Abstract

**Background:**

rhBMP-2 is regarded as the most potent osteoinductive growth factor, and it has been used in the oral cavity with different carriers. The purpose of this study is to evaluate the bone-regenerative effect of rhBMP-2 delivered with different carrier systems through three-dimensional cone beam computed tomography analysis.

**Method:**

A total of 112 patients underwent oral surgery with rhBMP-2 application (Group 1, *n* = 53) or without rhBMP-2 application (Group 2, *n* = 59). Group 1 was divided into 3 groups according to carriers, rhBMP-2 with allograft (Group 1–1, *n* = 34), rhBMP-2 with xenograft (Group 1–2, *n* = 5), and rhBMP-2 with absorbable collagen sponge (Group 1–3, *n* = 14). Cone beam computed tomography scans were taken before surgery (T0) 6 months after surgery (T1). The volume of defects was measured through the three-dimensional image analysis tool.

**Results:**

The average bone regeneration rate of Group 1 was significantly greater than that of Group 2. Within Group 1, the group that used allograft as a carrier (Group 1–1) showed significantly higher bone regeneration rates than the group that used absorbable collagen sponge as a carrier (Group 1–3).

**Conclusion:**

The use of rhBMP-2 after oral surgery results in a superior bone regeneration rate compared to not using rhBMP-2, and its efficacy depends on the carriers it is used with. Allograft affects bone regeneration more than absorbable collagen sponge when it is carried with rhBMP-2. Therefore, the appropriate use of rhBMP-2 with suitable bone grafting materials is useful for promoting postoperative bone regeneration in oral surgery.

## Background

Bone morphogenetic proteins (BMPs) regulate cellular processes such as differentiation, proliferation, and morphogenesis [1]. They are present within the bone and play a crucial role as inducers of bone formation in cases of bone fractures and bone defects caused by trauma or diseases [2]. The BMP family comprises approximately 40 known members, among which BMP-2 has been identified as the most potent stimulator of bone healing and bone formation [3, 4].

BMPs are present in small amounts within bone and do not exhibit species-specific characteristics. Therefore, extraction of BMPs from both homologous and heterologous sources has been extensively attempted to harness their osteogenic abilities. These proteins have been isolated from the mammalian bone, osteosarcoma, and dental pulp, among other sources [5, 6]. However, the extraction and purification of BMPs are complex and provide a limited yield [7]. Recent advancements in genetic engineering have made it possible to produce large quantities of BMPs through genetic manipulation.

More than 20 different types of recombinant human BMPs (rhBMPs) have been synthesized through genetic manipulation [8, 9]. These proteins can induce cartilage and bone formation and create a foundational environment for functional bone marrow formation during bone formation [10]. BMP-2 directly promotes nerve cell growth and induces chondrocyte and osteoblast differentiation. The differentiation of surrounding immature mesenchymal cells into osteogenic cells enables stimulation and regulation of bone formation [11–13].

Although BMPs have been commercialized and applied in clinical settings, they are currently available in liquid form. This poses challenges as BMPs alone are difficult to manipulate, have low stability, and tend to quickly diffuse and be absorbed and degraded in the body, making it difficult to induce bone formation effectively [14]. Therefore, the use of delivery systems is essential to overcome these limitations.

Carriers have been designed to control or slow down the release of BMPs while maintaining their biological activity, allowing for a sustained release at the desired time for bone formation and mitigating the initial burst release effect [15].

BMP carriers should be biocompatible, easily manipulated, and biodegradable in the body, without causing adverse tissue reactions. They should allow easy replacement with newly formed bone, maintain space for a specific period, and gradually release BMPs [16]. Through this, carriers should provide maximum opportunities for BMPs to come into contact with the surrounding cells [17, 18]. Various materials with these characteristics are being researched, and the most commonly used carriers include absorbable collagen sponge (ACS) and synthetic bone materials, such as hydroxyapatite or β-tricalcium phosphate, which may be used either alone or in combination.

Superior bone regeneration has been observed on applying BMPs with various carriers than that achieved without BMPs. However, the carrier demonstrating the most effective bone regeneration has not yet been conclusively identified [19, 20].

Therefore, we aimed to assess the effect of applying rhBMP-2 with different carriers, such as allografts, xenografts, and ACS, on bone regeneration by measuring the preoperative and postoperative volumes of maxillofacial bone defects through cone beam computed tomography (CBCT).

## Methods

### Patients

We included 112 patients diagnosed with cysts, benign tumors, or osteomyelitis of the jaw who underwent oral surgery at Ajou University Dental Hospital between 2019 and 2022. All patients were followed up for more than 6 months. Patients with uncontrolled systemic disease, infection, or lesion recurrence after surgery were excluded.

The patients who received rhBMP-2 (Novosis®, Dae-woong Bio, Korea) with different carriers at the defect site were assigned to the experimental group (Group 1, *n* = 53). Group 1 was divided into three sub-group according to the carrier used: allograft (Allomix®, CGBIO, Korea) (Group 1–1, *n* = 34), xenograft (The Graft®, PurgoBiologics, Korea) (Group 1–2, *n* = 5), and ACS (Ateloplug®, Hyundae Bioland, Korea) (Group 1–3, *n* = 14). Patients who did not receive rhBMP-2 were included in the control group (Group 2, *n* = 59). Group 2 included patients who received bone grafts (3 patients with allograft, 40 patients with xenograft) or ACS (13 patients) as well as those who received no material (3 patients).

This study was conducted with the approval of the Institutional Review Board of Ajou University Hospital (IRB No: AJOUIRB-DB-2023–201).

### CBCT examination and measurements

CBCT (Dinnova3, HDX, Korea) was performed preoperatively (T0) and at 6 months postoperatively (T1) at Ajou University Dental Hospital using the following imaging condition: 80kVp, 7.0 mA, and scan time for 20 s. The slice thickness was 0.3 mm, and the distance between slices was 1.0 mm. The digital CBCT scans were exported as Digital Imaging and Communications in Medicine (DICOM) format files to InVivo Dental Application version 5 (Anatomage, San Jose, CA).

### Bone regeneration rate analysis

**Fig. 1:**
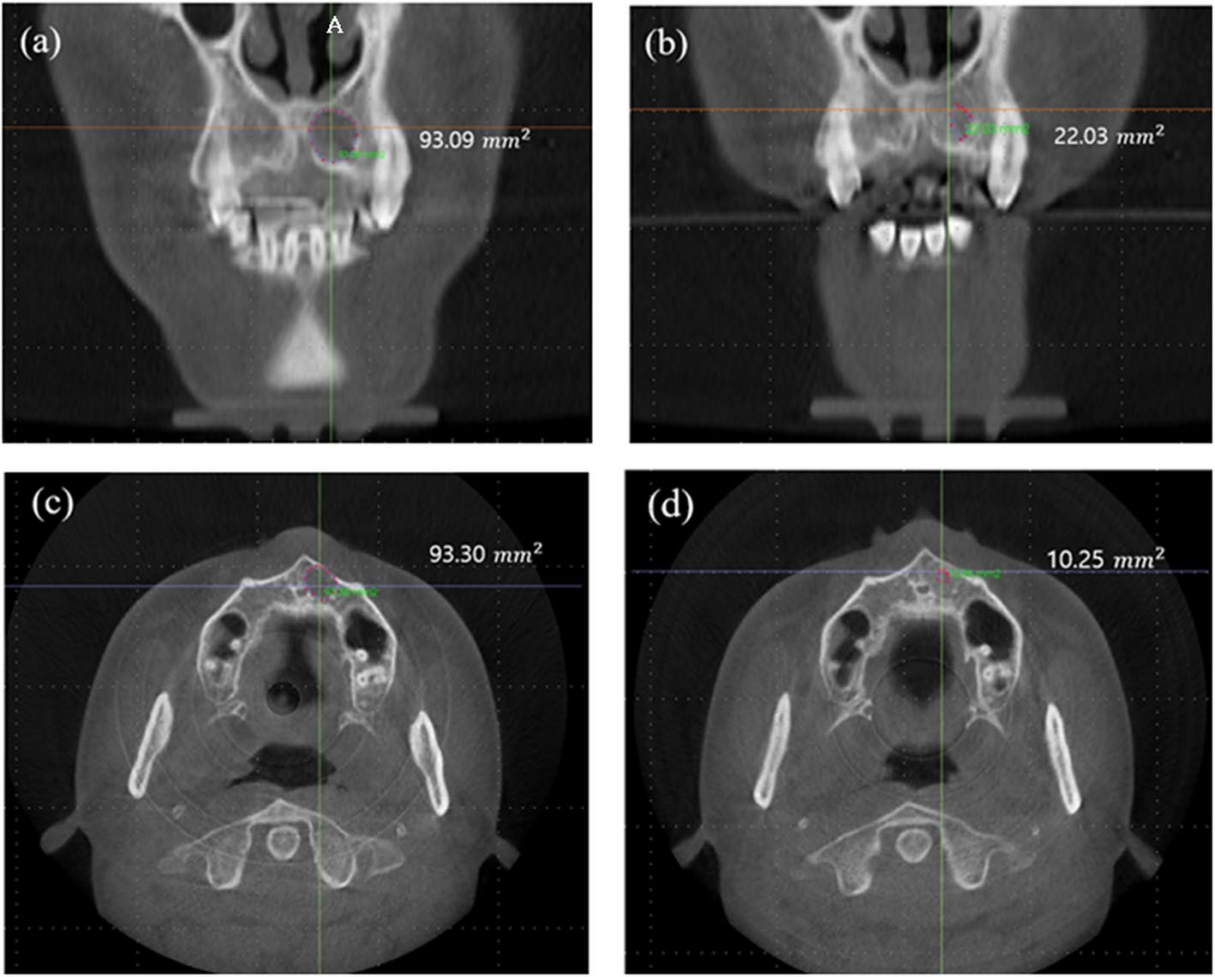
Preoperative and postoperative (6 months) defect size. Coronal view of preoperative defect size (**a**) and postoperative defect size (**b**) and axial view of preoperative defect size (**c**) and postoperative defect size (**d**). All patients showed a postoperative decrease in the area of the defect

**Fig. 2:**
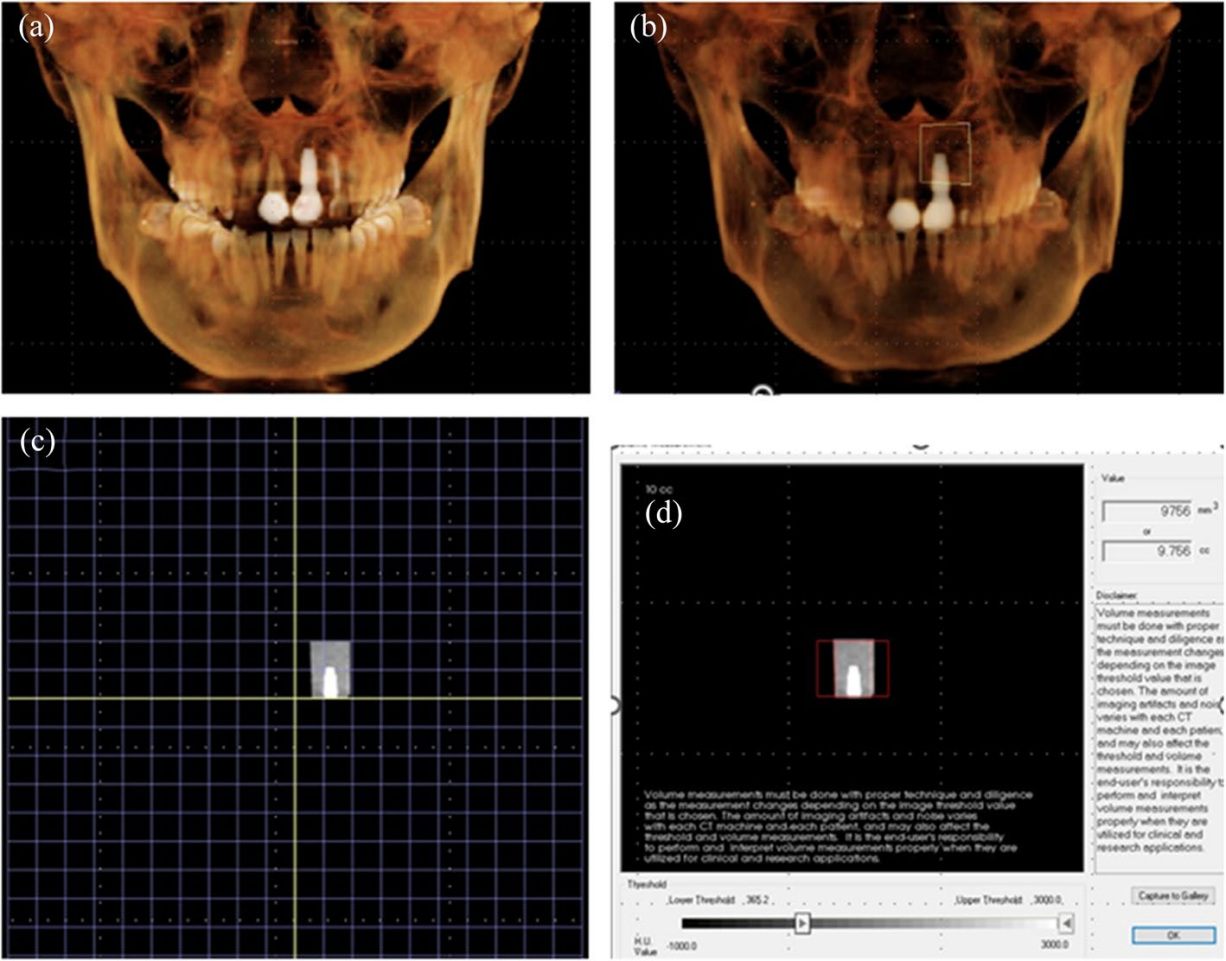
Defect bone volume measurement. After determining the regions of each defect (**a**, **b**), the functionality selects only the pixels within the range of the HU specified by the user (**c**). This enabled the calculation of volume for the selected pixels (**d**)

InVivo Dental Application version 5 was used to measure the volume (Fig. 1). After determining the regions of each defect, the functionality selects only the pixels within the range of the hounsfield unit (HU) specified by the user. This enabled the calculation of volume for the selected pixels (Fig. 2). Lesions with a density of -1000–200 HU were considered defects [21].

To minimize visual errors during volumetric measurements, the position of the patient’s head on the CBCT image was reoriented before measurement. Reorientation was performed in the coronal plane, and a line connecting both orbitales was used. In the horizontal plane, a line connecting the Frankfort horizontal plane passing through the orbitale and porion was used. In the sagittal plane, a line connecting the anterior and posterior nasal spines (PNS) was used.

The bone regeneration rate was calculated based on the preoperative and postoperative defect volumes as follows:

$$\begin{array}{l}\bf{Bone}\,\bf{regeneration}\,\bf{rate}\\=\;\frac{\left(pre\,-op\;defect\;bone\;volume\right)\;-\;\left(post\,-op\;defect\;bone\;volume\right)}{pre-op\;defect\;bone\;volume}\;\times\;100\;\end{array}$$

The measurements were performed twice with a 2-week washout period.

### Statistical analysis

A single investigator performed the measurements twice to minimize methodological errors. The Kolmogorov-Smirnova test was performed to test the normality of data. The Kruskal–Wallis, Mann–Whitney tests, and Bonferroni correction were performed to evaluate the bone regenerative rate in each group. Statistical significance was set at *p* < 0.05. SPSS Statistics version 25.0 (SPSS Inc., Chicago, IL, USA) was used for all statistical analyses.

## Result

The average age of all patients was 42.24 years, with 52 males and 60 females. In the experimental group where rhBMP-2 was used, there were a total of 53 patients with an average age of 46.47 years, including 28 males and 25 females. In the control group without the use of rhBMP-2, there were a total of 59 patients with an average age of 38.29 years, including 24 males and 35 females.

**Fig. 3:**
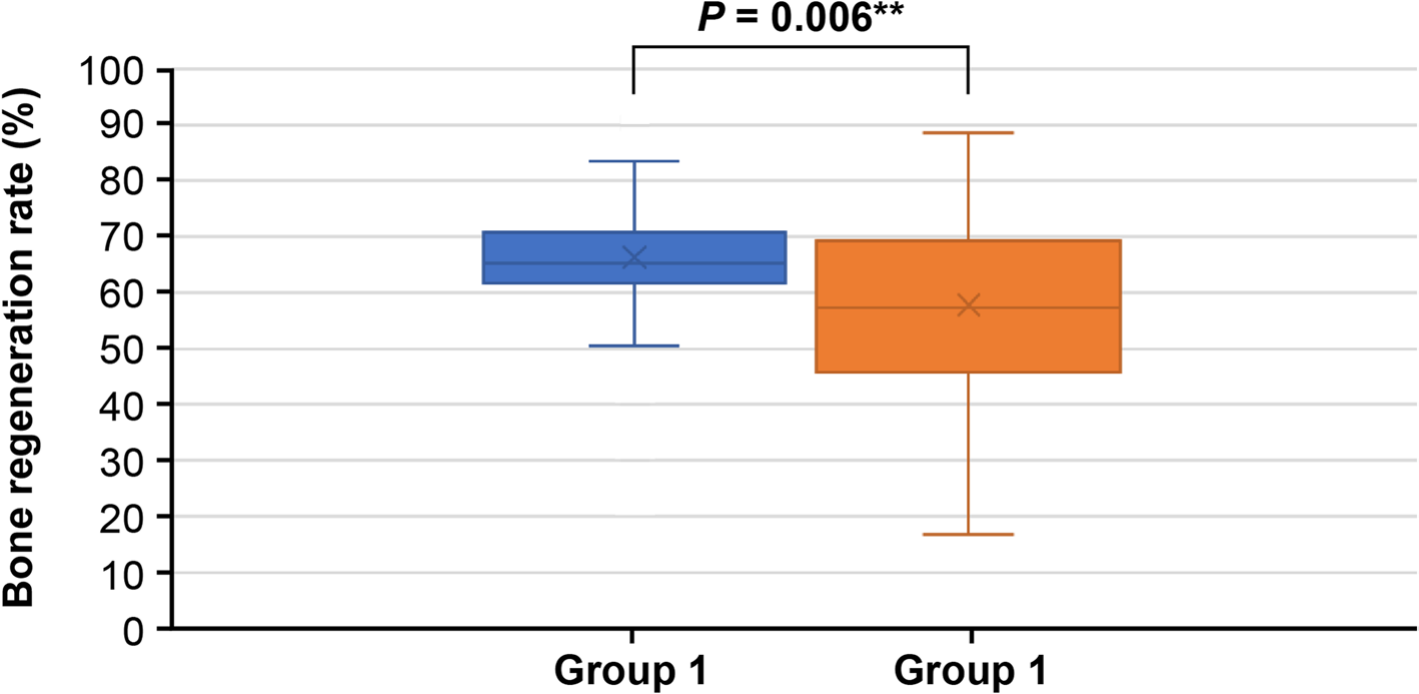
Bone regeneration rate of Group 1 and Group 2. The bone regeneration rate in the experimental group using rhBMP-2 (Group 1) was significantly higher compared to the control group without rhBMP-2 (Group 2)

All patients showed a decrease in the volume of the defect after surgery compared to before surgery. The bone regeneration rate in the experimental group, where rhBMP-2 was used, ranged from a minimum of 30.82% to a maximum of 89.38%, with an average of 66.28%. In the control group without the use of rhBMP-2, the bone regeneration rate ranged from a minimum of 16.72% to a maximum of 88.14%, with an average of 57.79%. The bone regeneration rate in the experimental group using rhBMP-2 was significantly higher compared to the control group without rhBMP-2 (Table 1, Fig. 3).

**Table 1:** Bone regeneration rate of Group 1 and Group 2

	**Bone regeneration rate**		***p* value**
	**Group 1 (*n* = 53)**	**Group 2 (*n* = 59)**	
Mean (sd)	66.3 (12.3)	57.8 (17.6)	0.006**
Median (IQR)	65.2 (61.9 to 70.6)	57.3 (45.8 to 69.2)	

The bone regeneration rate in the experimental group using rhBMP-2 (Group 1) was significantly higher compared to the control group without rhBMP-2 (Group 2)

*p* value from Mann–Whitney *U* test. Group 1, use rhBMP-2; Group 2, no use rhBMP-2. *IQR* Q1 to Q3

^*^p < 0.05

^**^p < 0.01

In the experimental group, the bone regeneration rate of Group 1–1 (using allograft as a carrier) ranged from a minimum of 60.22% to a maximum of 89.38%, with an average of 70.53%. The bone regeneration rate of Group 1–2 (using xenograft as a carrier) ranged from a minimum of 60.43% to a maximum of 70.43%, with an average of 65.13%. The bone regeneration rate of Group 1–3 (using ACS as a carrier) ranged from a minimum of 30.82% to a maximum of 85.92%, with an average of 56.36% (Table 2).

**Table 2:** Bone regeneration rate of Group 1–1, Group 1–2, and Group 1–3

	**Bone regeneration rate**			***p* value**
	**Group 1–1 (*n* = 34)**	**Group 1–2 (*n* = 5)**	**Group 1–3 (*n* = 14)**	
Mean (sd)	70.5 (9.1)	65.1 (4.2)	56.4 (15.2)	0.004**
Median (IQR)	67.4 (64.3 to 74.8)	64.6 (62.0 to 68.2)	56.5 (50.5 to 64.1)	

*p* value from kruskal–wallis test

Group 1–1, rhBMP-2 + allograft; Group 1–2, rhBMP-2 + xenograft; Group 1–3, rhBMP-2 + ACS. *IQR* Q1 to Q3

^*^p < 0.05

^**^p < 0.01

**Fig. 4:**
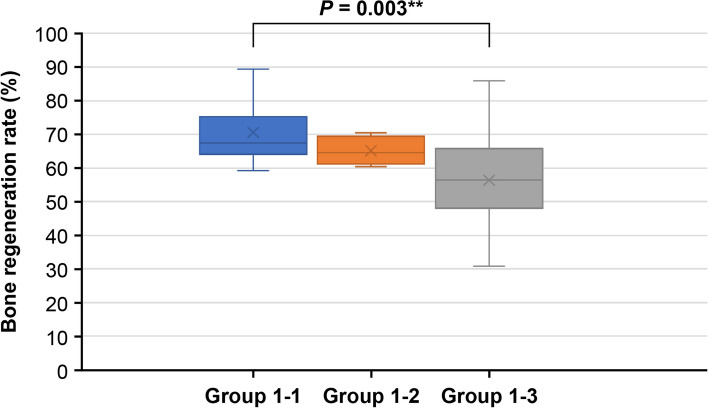
Bone regeneration rate of Group 1–1, Group 1–2, and Group 1–3. Group 1–1, which used allograft as a carrier showed a significantly higher bone regeneration rate compared to Group 1–3, which used ACS as a carrier

Group 1–1 which used allograft as a carrier showed a significantly higher bone regeneration rate compared to Group 1–3, which used ACS as a carrier (*p* = 0.003). However, there was no statistically significant difference in the bone regeneration rate between Group 1–2, which used xenograft as a carrier, and Group 1–3 (*p* = 0.803). Additionally, there was no statistically significant difference in the bone regeneration rate between Group 1–2 and Group 1–3 (*p* = 0.997) (Table 3, Fig. 4).

**Table 3:** Post hoc analysis comparing the mean of Group 1–1, Group 1–2, and Group 1–3

	**Mean rank difference**	***p* value†**
Group 1–1 with 1–2	− 8.2	0.803
Group 1–1 with 1–3	− 16.0	0.003**
Group 1–2 with 1–3	− 7.8	0.997

Group 1–1, which used allograft as a carrier showed a significantly higher bone regeneration rate compared to Group 1–3, which used ACS as a carrier

Multiple comparisons using rank sums: Bonferroni

Group 1–1, rhBMP-2 + allograft; Group 1–2, rhBMP-2 xenograft; Group 1–3, rhBMP-2 + ACS

†*p* value: adjusted *p* value (*p* = 0.05)

^*^p < 0.05

^**^p < 0.01

## Discussion

rhBMP-2 is a growth factor with excellent osteoinductive properties. It has therefore been actively studied [22, 23] and has received attention as a treatment method for bone defects. rhBMP-2 should be used with a carrier for maximum effectiveness, and the choice of carrier can influence the effects of rhBMP-2 [24].

The ideal carrier for rhBMP-2 should be biocompatible and capable of degrading at an appropriate rate while being replaced by newly formed bone. It should have porosity to allow the ingrowth of new blood vessels and cells. Most importantly, it should maintain BMP activity and enable controlled release at an appropriate rate [25, 26].

Although several studies have been conducted to identify ideal carriers, most have focused on animal models or histological analyses [27–29]. Therefore, in this study, we aimed to investigate the bone regeneration rate when rhBMP-2 is applied with various carriers through a three-dimensional analysis with CBCT. This approach allowed the identification of the most effective carrier for bone regeneration among allograft, xenograft, and ACS.

The present results revealed a significantly higher average bone regeneration rate when rhBMP-2 was applied to the site of bone defect after surgery than when rhBMP-2 was not applied. This finding aligns with those of previous studies demonstrating the osteogenic potential of rhBMP-2 [30, 31]. Furthermore, the choice of carrier material for rhBMP-2 also influenced the bone regeneration rate, which was significantly higher on using allograft bone as the carrier as compared with that on using ACS.

However, it is important to note that this was a retrospective analysis of patients who underwent surgery. The decision to use rhBMP-2 or the choice of carrier material was based on factors, such as the number of remaining walls in the bone defect, diagnosis, and patient age. In this study, in all patients with osteomyelitis, ACS with rhBMP-2 and carrier was applied to the area of the defect after surgery. In the case of osteomyelitis, rhBMP-2 was used for bone regeneration after removal of the sequestrum, and ACS was used together to reduce the risk of infection. For all other patients, there was no preference for a specific repair method. The number of compromised walls in patients with osteomyelitis was higher than that of patients with other diagnoses, and the existing defect volume of group 1–3 was also higher than that of other groups. These factors are potentially contributing to the lower bone regeneration rates in Group 1–3 compared to Group 1–1 and Group 1–2. Therefore, this study could not be conducted in a completely randomized manner, which may have influenced the bone regeneration rate findings (Tables 4 and 5).

**Table 4:** Epidemiological information on all patients

	Group 1 (*n* = 53)	Group 2 (*n* = 59)
Age (years)	46.47 ± 15.49	38.29 ± 18.03
Gender		
Male	28	24
Female	25	35
Diagnosis		
Cyst	7	21
Benign tumor	36	36
Osteomyelitis	6	0
Inflammation	4	2
Defect size (cc)	3.930 ± 2.282	3.345 ± 2.241
Compromised wall	1.25	1.28

**Table 5:** Epidemiological information on Group 1

	Group 1		
	Group 1–1 (*n* = 34)	Group 1–2 (*n* = 5)	Group 1–3 (*n* = 14)
Age (years)	44.86 ± 14.97	51.80 ± 9.73	48.71 ± 18.47
Gender			
Male	19	3	6
Female	15	2	8
Diagnosis			
Cyst	5	1	1
Benign tumor	27	2	7
Osteomyelitis	0	0	6
Inflammation	2	2	0
Defect size (cc)	3.569 ± 1.825	3.073 ± 0.981	5.162 ± 3.177
Compromised wall	1.07	1.2	1.79

Furthermore, there was a significant disparity in the sample size of the sub-group that received allografts as carriers (*n* = 34) and sub-group that received xenografts (*n* = 5). The small sample size of the xenograft group may have prevented a meaningful comparison of the two groups. Further studies with a larger sample are necessary to enable appropriate comparisons between these groups and obtain a comprehensive understanding.

However, this study has significant value because the CBCT scans of actual patients were evaluated preoperatively and at the 6-month follow-up postoperatively. This study provides meaningful insights into the effectiveness of rhBMP-2 in the clinical setting.

## Conclusion

This study aimed to investigate the impact of using rhBMP-2 with various carriers on bone regeneration in maxillofacial defects. The bone regeneration rate was calculated through a CBCT analysis computed to evaluate the effects of rhBMP-2. The use of rhBMP-2 after oral surgery resulted in a superior bone regeneration rate compared to that achieved without rhBMP-2, and its efficacy depended on the carrier used. Allograft carriers affected bone regeneration more than the ACS carrier. Therefore, the appropriate use of rhBMP-2 with suitable bone-grafting materials on CBCT analysis is useful to promote postoperative bone regeneration in oral surgery.

## Data Availability

The datasets used and analyzed during the current study are available from the corresponding author on reasonable request.

## Abbreviations

BMPs: Bone morphogenetic proteins
rhBMPs: Recombinant human bone morphogenetic proteins
ACS: Absorbable collagen sponge
CBCT: Cone beam computed tomography
HU: Hounsfield unit
PNS: Posterior nasal spine
